# Supplementary material for: Insurance coverage, stage at diagnosis, and time to treatment following dependent coverage and Medicaid expansion for men with testicular cancer
Source: PLoS One. 2020 Sep 16;15(9):e0238813. doi: 10.1371/journal.pone.0238813 (PMC7494102; doi:10.1371/journal.pone.0238813)
Supplement: S1 Table — a Comparisons based on Pearson’s Chi-squared analyses for discrete covariates and Mann-Whitney U test for age. (DOCX) [file pone.0238813.s001.docx]

**S1 Table:** Patient characteristics for Affordable Care Act Dependent Care Expansion analysis

| Characteristic | Age 19-25 years, n (%) | Age 26-64 years, n (%) | p^a^ |
| --- | --- | --- | --- |
| Total | 8,026 (100.0) | 33,303 (100.0) |  |
| Year |  |  |  |
| 2007 | 846 (10.5) | 3,572 (10.7) | 0.5 |
| 2008 | 852 (10.6) | 3,667 (11.0) |  |
| 2009 | 896 (11.2) | 3,622 (10.9) |  |
| 2011 | 910 (11.3) | 3,745 (11.3) |  |
| 2012 | 904 (11.3) | 3,858 (11.6) |  |
| 2013 | 936 (11.7) | 3,943 (11.8) |  |
| 2014 | 946 (11.8) | 3,962 (11.9) |  |
| 2015 | 925 (11.5) | 3,855 (11.6) |  |
| 2016 | 811 (10.1) | 3,079 (9.3) |  |
| Age, year |  |  |  |
| Median (IQR) | 23 (21-24) | 36 (30-44) | <0.001 |
| Race/Ethnicity |  |  |  |
| White | 5,615 (70.0) | 26,227 (78.8) | <0.001 |
| Black | 196 (2.4) | 1,118 (3.4) |  |
| Hispanic | 1,494 (18.6) | 3,098 (9.3) |  |
| Unknown/other | 721 (9.0) | 2,860 (8.6) |  |
| Comorbidities |  |  |  |
| 0 | 7,643 (95.2) | 30,887 (92.8) | <0.001 |
| 1 | 360 (4.5) | 2,023 (6.1) |  |
| >1 | 23 (0.3) | 393 (1.2) |  |
| Income |  |  |  |
| ≤$40,227 | 1,326 (16.5) | 4,675 (14.0) | <0.001 |
| $40,227-50,353 | 1,718 (21.4) | 6,592 (19.8) |  |
| $50,354-63,332 | 1,974 (24.6) | 7,993 (24.0) |  |
| ≥$63,000 | 3,008 (37.5) | 14,043 (42.2) |  |
| Non-high school educated in patient's zip code |  |  |  |
| ≥17.6% | 1,869 (23.3) | 5,884 (17.7) | <0.001 |
| 10.9-17.5% | 1,799 (22.4) | 7,730 (23.2) |  |
| 6.3-10.8% | 2,158 (26.9) | 9,714 (29.2) |  |
| ≤6.3% | 2,200 (27.4) | 9,975 (30.0) |  |
| No insurance | 1,185 (14.8) | 3,414 (10.3) | <0.001 |
| Stage at diagnosis ≥II | 2,904 (36.2) | 9,106 (27.3) | <0.001 |
| In those with orchiectomy as first treatment, treatment 14 days or more after diagnosis |  |  |  |
| No | 3,312 (91.2) | 13,852 (90.2) | 0.066 |
| Yes | 321 (8.8) | 1,511 (9.8) |  |
| In those with chemotherapy or radiotherapy as first treatment, treatment 60 days or more after diagnosis |  |  |  |
| No | 2,727 (78.6) | 8,822 (79.3) | 0.400 |
| Yes | 742 (21.4) | 2,307 (20.7) |  |

^a^ Comparisons based on Pearson’s Chi-squared analyses for discrete covariates and Mann-Whitney U test for age.
