# Supplementary material for: Insurance coverage, stage at diagnosis, and time to treatment following dependent coverage and Medicaid expansion for men with testicular cancer
Source: PLoS One. 2020 Sep 16;15(9):e0238813. doi: 10.1371/journal.pone.0238813 (PMC7494102; doi:10.1371/journal.pone.0238813)
Supplement: S2 Table — These data were used to generate Table 1 and Fig 1. (DOCX) [file pone.0238813.s002.docx]

**S2 Table:** Raw data for difference-in-difference analyses for ACA-DCE

| Outcome | Year of diagnosis | Age 19-25 years | | Age 26-64 years | |
| --- | --- | --- | --- | --- | --- |
|  |  | Total, n | Yes, n (%) | Total, n | Yes, n (%) |
| No insurance | 2007 | 846 | 165 (19.5) | 3572 | 326 (9.1) |
|  | 2008 | 852 | 150 (17.6) | 3667 | 376 (10.3) |
|  | 2009 | 896 | 162 (18.1) | 3622 | 394 (10.9) |
|  | 2011 | 910 | 152 (16.7) | 3745 | 475 (12.7) |
|  | 2012 | 904 | 149 (16.5) | 3858 | 439 (11.4) |
|  | 2013 | 936 | 128 (13.7) | 3943 | 507 (12.9) |
|  | 2014 | 946 | 111 (11.7) | 3962 | 356 (9) |
|  | 2015 | 925 | 98 (10.6) | 3855 | 309 (8) |
|  | 2016 | 811 | 70 (8.6) | 3079 | 232 (7.5) |
|  |  | Total, n | Yes, n (%) | Total, n | Yes, n (%) |
| Stage at diagnosis ≥II | 2007 | 846 | 283 (33.5) | 3572 | 864 (24.2) |
|  | 2008 | 852 | 292 (34.3) | 3667 | 894 (24.4) |
|  | 2009 | 896 | 309 (34.5) | 3622 | 934 (25.8) |
|  | 2011 | 910 | 315 (34.6) | 3745 | 1056 (28.2) |
|  | 2012 | 904 | 323 (35.7) | 3858 | 1047 (27.1) |
|  | 2013 | 936 | 334 (35.7) | 3943 | 1080 (27.4) |
|  | 2014 | 946 | 345 (36.5) | 3962 | 1096 (27.7) |
|  | 2015 | 925 | 349 (37.7) | 3855 | 1101 (28.6) |
|  | 2016 | 811 | 354 (43.6) | 3079 | 1034 (33.6) |
|  |  | Total, n | Yes, n (%) | Total, n | Yes, n (%) |
| In those with orchiectomy as first treatment, treatment 14 days or more after diagnosis | 2007 | 378 | 33 (8.7) | 1310 | 113 (8.6) |
|  | 2008 | 338 | 31 (9.2) | 1399 | 111 (7.9) |
|  | 2009 | 369 | 28 (7.6) | 1389 | 140 (10.1) |
|  | 2011 | 411 | 28 (6.8) | 1591 | 152 (9.6) |
|  | 2012 | 413 | 38 (9.2) | 1813 | 179 (9.9) |
|  | 2013 | 451 | 32 (7.1) | 1986 | 207 (10.4) |
|  | 2014 | 468 | 41 (8.8) | 2102 | 214 (10.2) |
|  | 2015 | 451 | 46 (10.2) | 2128 | 208 (9.8) |
|  | 2016 | 354 | 44 (12.4) | 1645 | 187 (11.4) |
|  |  | Total, n | Yes, n (%) | Total, n | Yes, n (%) |
| In those with chemotherapy or radiotherapy as first treatment, treatment 60 days or more after diagnosis | 2007 | 312 | 67 (21.5) | 874 | 193 (22.1) |
|  | 2008 | 357 | 97 (27.2) | 1046 | 204 (19.5) |
|  | 2009 | 393 | 97 (24.7) | 1143 | 233 (20.4) |
|  | 2011 | 389 | 68 (17.5) | 1265 | 260 (20.6) |
|  | 2012 | 402 | 84 (20.9) | 1372 | 281 (20.5) |
|  | 2013 | 396 | 73 (18.4) | 1424 | 290 (20.4) |
|  | 2014 | 407 | 86 (21.1) | 1427 | 264 (18.5) |
|  | 2015 | 413 | 86 (20.8) | 1380 | 315 (22.8) |
|  | 2016 | 400 | 84 (21) | 1198 | 267 (22.3) |

These data were used to generate Table 1 and Figure 1.
