# Supplementary material for: Insurance coverage, stage at diagnosis, and time to treatment following dependent coverage and Medicaid expansion for men with testicular cancer
Source: PLoS One. 2020 Sep 16;15(9):e0238813. doi: 10.1371/journal.pone.0238813 (PMC7494102; doi:10.1371/journal.pone.0238813)
Supplement: S3 Table — Multivariable linear regression analyses were used to evaluate difference-in-differences for each outcome between intervention and controls, and pre- (2007–2009) and individual post-exposure years (2011–2016). Bolded p values are statistically significant (p < 0.05). Abbreviation: ACA-DCE, Affordable Care Act Dependent Care Expansion; CI, confidence interval. (DOCX) [file pone.0238813.s003.docx]

| **S3 Table:** Difference-in-difference analyses on outcomes for men with testicular cancer following ACA-DCE. | | | | | | | | | | | | |
| --- | --- | --- | --- | --- | --- | --- | --- | --- | --- | --- | --- | --- |
|  | Difference in differences of pre-expansion (2007-2009) vs. individual years | | | | | | | | | | | |
|  | 2011 | p | 2012 | p | 2013 | p | 2014 | p | 2015 | p | 2016 | p |
| % Men without insurance coverage | | | | | | | | | | | | |
| All men | -4.80 | **<0.001** | -2.23 | 0.089 | -7.26 | **<0.001** | -5.86 | **<0.001** | -6.11 | **<0.001** | -7.43 | **<0.001** |
| Regional low-income | -2.14 | 0.6 | -2.11 | 0.6 | -8.31 | 0.060 | -3.46 | 0.4 | -4.78 | 0.2 | -6.45 | 0.146 |
| % Men with advanced stage at diagnosis | | | | | | | | | | | | |
| All men | -3.02 | 0.118 | -0.14 | 0.9 | -0.89 | 0.6 | -0.45 | 0.8 | -0.16 | 0.9 | 0.97 | 0.6 |
| Regional low-income | -3.55 | 0.5 | 0.16 | 0.9 | -2.57 | 0.6 | -2.96 | 0.6 | 5.72 | 0.2 | -1.18 | 0.8 |
| % Men whose first treatment was orchiectomy who received treatment greater than 14 days after diagnosis | | | | | | | | | | | | |
| All men | -2.27 | 0.2 | -0.09 | 0.9 | -2.85 | 0.122 | -1.01 | 0.6 | 0.84 | 0.6 | 1.67 | 0.4 |
| Regional low-income | 1.47 | 0.8 | 4.94 | 0.4 | 1.74 | 0.7 | 3.41 | 0.5 | 1.78 | 0.7 | 2.52 | 0.6 |
| % Men whose first treatment was chemotherapy or radiotherapy who received treatment greater than 60 days following diagnosis | | | | | | | | | | | | |
| All men | -7.26 | **0.009** | -3.26 | 0.2 | -5.96 | **0.029** | -1.46 | 0.6 | -6.1 | **0.024** | -5.38 | 0.051 |
| Regional low-income | -1.17 | 0.9 | -3.37 | 0.6 | -9.26 | 0.2 | -5.42 | 0.4 | -5.34 | 0.4 | -0.41 | 0.9 |
| Multivariable linear regression analyses were used to evaluate difference-in-differences for each outcome between intervention and controls, and pre- (2007-2009) and individual post-exposure years (2011-2016). Bolded p values are statistically significant (p < 0.05); CI, confidence interval. | | | | | | | | | | | | |
