# Supplementary material for: Insurance coverage, stage at diagnosis, and time to treatment following dependent coverage and Medicaid expansion for men with testicular cancer
Source: PLoS One. 2020 Sep 16;15(9):e0238813. doi: 10.1371/journal.pone.0238813 (PMC7494102; doi:10.1371/journal.pone.0238813)
Supplement: S4 Table — a Comparisons based on Pearson’s Chi-squared analyses for discrete covariates and Mann-Whitney U test for age. (DOCX) [file pone.0238813.s004.docx]

**S4 Table:** Patient characteristics for Medicaid Expansion analysis

| Characteristic | Expansion, n (%) | Non-expansion, n (%) | p ^a^ |
| --- | --- | --- | --- |
| Total | 2,296 (100.0) | 2,265 (100.0) |  |
| Year |  |  |  |
| 2011 | 517 (22.5) | 481 (21.2) | 0.124 |
| 2012 | 507 (22.1) | 485 (21.4) |  |
| 2013 | 494 (21.5) | 469 (20.7) |  |
| 2015 | 428 (18.6) | 494 (21.8) |  |
| 2016 | 350 (15.2) | 336 (14.8) |  |
| Age, year |  |  |  |
| Median (IQR) | 47 (43-53) | 47 (43-52) | 0.3 |
| Race/Ethnicity |  |  |  |
| White | 2,010 (87.5) | 1,860 (82.1) | <0.001 |
| Black | 58 (2.5) | 143 (6.3) |  |
| Hispanic | 83 (3.6) | 141 (6.2) |  |
| Unknown/other | 145 (6.3) | 121 (5.3) |  |
| Comorbidities |  |  |  |
| 0 | 2,034 (88.6) | 1,991 (87.9) | 0.5 |
| 1 | 210 (9.2) | 211 (9.3) |  |
| >1 | 52 (2.3) | 63 (2.8) |  |
| Income |  |  |  |
| ≤$40,227 | 268 (11.7) | 436 (19.3) | <0.001 |
| $40,227-50,353 | 424 (18.5) | 562 (24.8) |  |
| $50,354-63,332 | 471 (20.5) | 519 (22.9) |  |
| ≥$63,000 | 1,133 (49.4) | 748 (33.0) |  |
| Non-high school educated in patient's zip code |  |  |  |
| ≥17.6% | 308 (13.4) | 483 (21.3) | <0.001 |
| 10.9-17.5% | 529 (23.0) | 579 (25.6) |  |
| 6.3-10.8% | 656 (28.6) | 603 (26.6) |  |
| ≤6.3% | 803 (35.0) | 600 (26.5) |  |
| No insurance | 146 (6.4) | 290 (12.8) | <0.001 |
| Stage at diagnosis ≥II | 647 (28.2) | 704 (31.1) | 0.032 |
| In those with orchiectomy as first treatment, treatment 14 days or more after diagnosis |  |  |  |
| No | 996 (86.8) | 949 (88.7) | 0.183 |
| Yes | 151 (13.2) | 121 (11.3) |  |
| In those with chemotherapy or radiotherapy as first treatment, treatment 60 days or more after diagnosis |  |  |  |
| No | 588 (78.4) | 657 (79.6) | 0.5 |
| Yes | 162 (21.6) | 166 (20.4) |  |

^a^ Comparisons based on Pearson’s Chi-squared analyses for discrete covariates and Mann-Whitney U test for age.
