# Supplementary material for: Insurance coverage, stage at diagnosis, and time to treatment following dependent coverage and Medicaid expansion for men with testicular cancer
Source: PLoS One. 2020 Sep 16;15(9):e0238813. doi: 10.1371/journal.pone.0238813 (PMC7494102; doi:10.1371/journal.pone.0238813)
Supplement: S5 Table — These data were used to generate Table 1 and Fig 2. (DOCX) [file pone.0238813.s005.docx]

**S5 Table:** Raw data for difference-in-difference analyses for Medicaid expansion

| Outcome | Year of diagnosis | Expansion | | Non-expansion | |
| --- | --- | --- | --- | --- | --- |
|  |  | Total, n | Yes, n (%) | Total, n | Yes, n (%) |
| No insurance | Pre-expansion | 1,518 | 131 (8.6) | 1435 | 198 (13.8) |
|  | Post-expansion | 778 | 15 (1.9) | 830 | 92 (11.1) |
|  |  | Total, n | Yes, n (%) | Total, n | Yes, n (%) |
| Stage at diagnosis ≥II | Pre-expansion | 1,518 | 412 (27.1) | 1435 | 425 (29.6) |
|  | Post-expansion | 778 | 235 (30.2) | 830 | 279 (33.6) |
|  |  | Total, n | Yes, n (%) | Total, n | Yes, n (%) |
| In those with orchiectomy as first treatment, treatment 14 days or more after diagnosis | Pre-expansion | 699 | 94 (13.4) | 640 | 63 (9.8) |
|  | Post-expansion | 448 | 57 (12.7) | 430 | 58 (13.5) |
|  |  | Total, n | Yes, n (%) | Total, n | Yes, n (%) |
| In those with chemotherapy or radiotherapy as first treatment, treatment 60 days or more after diagnosis | Pre-expansion | 488 | 108 (22.1) | 503 | 87 (17.3) |
|  | Post-expansion | 262 | 54 (20.6) | 320 | 79 (24.7) |

These data were used to generate Table 1 and Figure 2
