# Supplementary material for: Insurance coverage, stage at diagnosis, and time to treatment following dependent coverage and Medicaid expansion for men with testicular cancer
Source: PLoS One. 2020 Sep 16;15(9):e0238813. doi: 10.1371/journal.pone.0238813 (PMC7494102; doi:10.1371/journal.pone.0238813)
Supplement: Attachment — Submitted filename: Response to Reviewers.docx [file pone.0238813.s006.docx]

July 20, 2020

Joerg Heber, PhD and Ernest K. Amankwah, PhD

PLOS One

**Re: Manuscript ID PONE-D-20-10591**

Dear Dr. Heber and Dr. Amankwah:

Please accept for review our revised manuscript entitled, **“Insurance coverage, stage at diagnosis, and time to treatment following dependent coverage and Medicaid expansion for men with testicular cancer.”** We appreciate the thoughtful and instructive comments from our reviewers, and have made the following changes (**all changes to our manuscript are bolded in this document and tracked changes/highlighted in the marked copy of the manuscript).** Line numbers refer to the “marked” manuscript.

**Reviewer responses**

**Academic editor:**

Address the following comments in addition to the reviewers’ comments

Abstract

1) The n’s in the methods of the abstract are a bit confusing. For example, it is not clear if n=41,329 refers to only the post-population. Revise for clarity

**Reply**

We agree the two sample sizes in the abstract were vague. We have updated the wording such that it is clearer the two sample sizes refer to the total sample sizes for the ACA-DCE analysis and Medicaid expansion analysis.

**Line 26**

Using a US-based cancer registry, we performed adjusted difference-in-difference (DID) analyses comparing outcomes between men aged 19-25 **(n=8,026)** and 26-64 **(n=33,303)** pre- (2007-2009) and post-ACA-DCE (2011-2016) and between men in states that expanded Medicaid **(n=2,296)** to men in those that did not **(n=2,265)** pre- (2011-2013) and post-Medicaid expansion (2015-2016).

2) In the results of the abstract, clarify when results are for all men and when they are comparing 19-25 and 26+

**Reply**

We agree the percentage changes in the abstract were not clearly referring to the difference-in-differences. We have updated the abstract to make it clearer the percentages are all referring to difference-in-differences.

**Line 34**

In ACA-DCE analysis, rates of uninsurance decreased **(DID -5.64, 95%** confidence interval [CI] -7.23 to -4.04%, p<0.001) among patients aged 19-25 relative to older patients aged 26-64. There was no significant **DID in advanced stage at diagnosis** (stage≥II; p=0.6) nor orchiectomy more than 14 days after diagnosis (p=0.6). For patients who received chemotherapy or radiotherapy as their first course of treatment, **treatment greater than 60 days after diagnosis decreased (DID -4.84%,** 95% CI -8.22 to -1.45%, p=0.005) **among patients aged 19-25 relative to patients aged 26-64**. In Medicaid expansion states, rates of uninsurance decreased (DID -4.20%, 95% CI -7.67 to -0.73%, p=0.018) while patients receiving chemotherapy or radiotherapy greater than 60 days after diagnosis decreased (DID -8.76, 95% CI -17.13 to -0.38%, **p=0.040**) **compared to rates in non-expansion states.** No significant DIDs were seen for stage (p=0.8) nor time to orchiectomy (p=0.1).

3) Be consistent with the decimal places for the non-significant p-values in the abstract. I will suggest you change p=0.109 to p=0.1

**Reply**

We agree that consistency would improve the readability of the manuscript and have updated the paper in several locations for these adjustments. We have kept three decimal places for non-significant p values that are <0.1. For instance, we have left p=0.080 as is.

Methods

4) Line 120: Move n=49221,100% to line 119 after “all men”

**Reply**

We agree that, similar to 1 from this reviewer that this change more clearly designates what this sample size references.

**Line 97**

For the analysis of the ACA-DCE, we included all male patients **(n=49,221, 100%)** diagnosed with testicular cancer ages 19-64 from 2007 to 2009 and 2011 to 2016.

5) Line 126: Move n=5601,100% to line 123 after “all men”

**Reply**

As in the previous comment, we agree this adds clarity to the reference for this sample size.

**Line 101**

For the Medicaid expansion analysis, we included all male patients **(n=5,601, 100%)** diagnosed with testicular cancer ages 40-64 from 2011 to 2013 and 2015 to 2016 residing in states that either expanded Medicaid on January 1, 2014 or never expanded Medicaid.

6) Lines 126-127 somehow conflicts with line 124. Revise one of these sentences as you cannot include 19-64 years if the database has information for men aged 40+ only

**Reply**

We agree this was an error on our part and we have corrected the error. The Medicaid expansion analysis was limited to men aged 40 to 64.

**Line 101**

For the Medicaid expansion analysis, we included all male patients (n=5,601, 100%) diagnosed with testicular cancer ages **40**-64 from 2011 to 2013 and 2015 to 2016 residing in states that either expanded Medicaid on January 1, 2014 or never expanded Medicaid. In this analysis, there was only data available in the NCDB on Medicaid expansion for patients age 40 years or older.

7) Provide a rationale for selecting 14 days and 60 days for orchiectomy and chemotherapy or radiation respectively

**Reply**

To the reviewer’s point, there is no specific guideline on the optimal timing for treatment for men with testicular cancer. Thus, the cutoffs for timing of orchiectomy and chemotherapy or radiation were chosen *a priori* to reflect favorable (earlier treatment) versus unfavorable (delayed treatment) access to healthcare. We now make this point clearer in the methods.

**Line 128**

**In the absence of specific guidelines on treatment timing, the cutoffs for timing of treatment were chosen *a priori* as generally acceptable timeframes as reflections of favorable (earlier treatment) versus unfavorable (delayed treatment) access to healthcare.**

8) Provide a rationale for comparing the two different age groups

**Reply**

To add clarity to the rationale for the age groups, we have updated the methods. The age groups were chosen on the ages of men who would have benefitted from ACA-DCE (ages 19-25) and those who wouldn’t benefit from ACA-DCE and would be too young for Medicare (ages 26-64).

**Line 142**

Controls were those aged 26-64 **as this age group would not have been affected by the ACA-DCE and would have been too young to receive Medicare**.^1^

Results

9) Lines 180-183: Clarify the comparison groups. If you are comparing 19-25 to 26-64 then it is obvious that they will be younger. You could instead indicate that “the median age for 19-24 group was …”

**Reply**

We agree and have adjusted this section as the reviewer suggested.

**Line 164**

**The median age at diagnosis for the patients age 19-25 years was 23 years vs 36 for patients age 26-64 years (p<0.001)**.

10) Provide the percentage of men that received orchiectomy as first line of therapy

**Reply**

We have provided these percentages more clearly in the results section.

**Line 169**

Among those with information on treatment type and timing of treatment (n=33,594), **18,996 (57%) received orchiectomy as their first treatment while** 14,598 (43%) received chemotherapy or radiotherapy as their first form of treatment.

11) Provide the pre and post percentages for each age group in Table 1 in addition to the DID or in a different table

**Reply**

We agree that this sort of supplemental data will improve the study transparency. We have created two new supplemental tables which now show percentages and pre- and post-intervention raw numbers and percentages which were used to generate DIDs and the two figures. These are the new S2 Table and S5 Table.

Discussion

12) I will not refer to this pre and post study as a cohort study

**Reply**

We agree, the term is not used correctly here. We have removed the word “cohort.”

**Line 277**

In this retrospective study…

**Reviewer #1**

Summary: The authors conducted a retrospective cohort study that investigated changes in rates of insurance coverage, cancer stage at diagnosis, and time to treatment among men with testicular cancer following introduction of the Affordable Care Act’s Dependent Care Expansion (ACA-DCE) in 2010 and the Medicare expansion program in 2014. Data were obtained from the National Cancer Database (NCDB). Because the ACA-DCE permits dependent coverage for adults between the ages 19 to 25 years, participants were considered exposed to the “intervention” if they were between 19 to 25 years at the time of diagnosis, while those between the ages 26 to 64 years at diagnosis were used as controls. For the Medicaid expansion analysis, patients were considered exposed to the “intervention” if they resided in a state that expanded Medicaid on January 1, 2014, while those who resided in states that did not adopt the Medicare expansion coverage were used as controls. For statistical analysis, multivariable-adjusted linear regression was used. The results show that compared to the control group, ACA-DCE beneficiaries of age 19 to 25 and patients in Medicaid expansion states experienced significant increases in medical insurance rates following ACA-DCE in 2010 and Medicaid expansion in 2014. These two groups also experienced a decrease in time to treatment for those whose first-line treatment was chemotherapy or radiotherapy as compared with controls, but no differences were observed in stage at diagnosis or time to treatment for those whose first-line therapy was orchiectomy. Overall, this study adds to the literature on the impact of ACA-DCE and the Medicare expansion program on health outcomes. Below are few suggestions for improvement.

1) For the ACA-DCE analysis, patients in the control group are older than those in the control group. This raises the concern of whether the observed differences could have been driven by age and not necessary the introduction of ACA-DCE. I wonder why the authors didn’t choose a control group of similar age range as the intervention group. Could the authors have used testicular cancer patients between age 19 to 25 years who did not benefit from ACA-DCE as controls?

**Reply**

This is a similar point to that made by the Academic Editor comment #8. We have updated our methods section to be clearer about why the age group 19 to 25 years was compared to the age group 26 to 64 years. In short, the ACA-DCE would apply to all men age 19 to 25 years so there would be no men in that age to whom it would not apply. Thus, the only “control” group had to be older men. We acknowledge that even though age was included in our multivariable linear regression which generated the difference-in-difference values, the age discrepancies between age groups may bias results. This is now noted in the limitations.

**Line 140**

In the analysis of the ACA-DCE, patients were considered to be exposed to the “intervention” if they were age 19-25 at the time of diagnosis**. Controls were those aged 26-64 as this age group would not have been affected by the ACA-DCE and would have been too young to receive Medicare**.^1^

**Line 352**

**Additionally, unbalanced age at diagnosis between in the comparison groups in the ACA-DCE analysis may create inherent bias given all patients age 19 to 25 years would have benefited from ACA-DCE, thus an older “control” group was required.**

2) Abstract: Please make explicitly clear which patients belonged to the “intervention” group versus the comparison groups. It is quite confusing to understand until I read the entire manuscript.

**Reply**

This is a similar point to that made by the Academic Editor comment #1. We have adjusted the abstract to clarify the sample sizes for each analysis and “intervention” versus “control” groups. We made similar adjustments in the results section for added clarity as well.

**Line 26**

Using a US-based cancer registry, we performed adjusted difference-in-difference (DID) analyses comparing outcomes between men aged 19-25 **(n=8,026)** and 26-64 **(n=33,303)** pre- (2007-2009) and post-ACA-DCE (2011-2016) and between men in states that expanded Medicaid **(n=2,296)** to men in those that did not **(n=2,265)** pre- (2011-2013) and post-Medicaid expansion (2015-2016).

3) Abstract: In the concluding statement, please indicate that two of the hypotheses tested produced null results; i.e., no association between ACA-DCE coverage and advanced state at diagnosis or orchiectomy.

**Reply**

We agree that highlighting null results are equally valuable in the case of this study and most studies. We have made the suggested adjustment to the abstract.

**Line 50**

ACA-DCE and Medicaid expansions **were associated with lower uninsurance rates and decreased time to delivery of chemotherapy or radiotherapy among men with testicular cancer. Time to orchiectomy and stage at diagnosis did not change following either insurance expansion.**

4) Methods: Stratified analysis by race/ethnicity would be of great interest as it will show whether the findings are consistent across racial minority groups or not.

**Reply**

This is an excellent point made by the reviewer. We had previously planned to do an exploratory analysis by race/ethnicity. However, with White patients comprising over 70% of the ACA-DCE analysis and over 80% of the Medicaid expansion analysis, the numbers of Black and Hispanic men were quite limited for an already relatively rare cancer. For the reviewer’s information, the findings for each outcome and expansion were non-significant for Black men, Hispanic men, and Black and Hispanic men combined (all p>0.1). However, due to the small numbers of these patients, we felt including these analyses may be mis-interpreted as suggesting insurance expansion benefited White patients more than those of Black or Hispanic race/ethnicity. For this reason, we hope the reviewer agrees this comment reflects a limitation of the dataset which we now acknowledge in our limitation section.

**Line 358**

**The current study was also limited by relatively small sample sizes and short follow-up leading to reduced patient numbers for subgroup analyses. For this reason, ongoing work should continue to evaluate the differential effects of insurance expansion for vulnerable patient groups based on race and ethnicity.**

5) The repeated use of “men” in the results section presupposes that women may have been included. Since testicular cancer occurs only in men, I suggest stating briefly in the first paragraph of results section that only men were included in the study and then reduce the use of “men” throughout this section.

**Reply**

We agree with this point and have made clear in the methods section that only “male patients” were included and have substantially minimized the frequent repetition of “men” in the results and discussion.

**Line 98**

For the analysis of the ACA-DCE, we included **all male patients** (n=49,221, 100%) diagnosed with testicular cancer ages 19-64 from 2007 to 2009 and 2011 to 2016.

6) Study limitations: Please include residual confounding by poorly measured factors (e.g., neighborhood income level and neighborhood education level, as opposed to individual level data) and confounding by unmeasured factors, such as smoking history, BMI, etc.

**Reply**

We agree and have updated our limitation to reflect this comment.

**Line 356**

**Several unmeasured relevant variables not included in the NCDB that may have affected our outcomes warrant mentioning including individual-level measurements of socioeconomic status, smoking history, and body-mass index among others.**

**Reviewer #2:**

Thank you for the opportunity to review this manuscript. The purpose of the paper was to assess the impact of ACA-DCE on adults aged 19-25 and Medicaid expansion on outcomes for men with testicular cancer. The authors found rates of uninsurance decreased among men aged 19-25 relative to older men, and in Medicaid expansion states, rates of uninsurance also decreased. The authors provided valuable information that is significant to oncology regarding the relationship between expanded coverage and outcomes in testicular cancer in adolescent and young males. Overall, the manuscript is well organized, and the authors' data and analyses fully supported their hypotheses and provided statistical measures that captured the exposure to insurance expansion. I recommend the manuscript for publication with the following minor recommendations:

1) Introduction – The authors provided appropriate evidence to support their claim for their study. However, in the introduction (line 103), it is mentioned that this study is the first to be studied in adolescent and young adult males. Are there any prior studies in other areas of oncology and young adults to boost the importance of expanded coverage and delayed treatment in this population?

**Reply**

This is an important point from this reviewer. There are previous works on the topic of cancer patients and insurance expansion. Most of these studies assessed rates of insurance coverage for all cancer patients. There are few examples of studies assessing the association between individual tumors types and outcomes specific to those tumor types and the current works available have shown differential effect of insurance expansion on young patients depending on the cancer type. We now cite these papers and make the point clearer that no previous work has looked at relevant outcomes specific to testicular cancer in the setting of insurance expansion. We believe the addition has improved our presentation of the relevance of the study in the introduction.

**Line 76**

**Previous works have assessed the associations between insurance expansion and outcomes specific to certain tumor types such as prostate and breast cancer,^2, 3^ which have shown differential benefits of insurance expansion for young patients with cancer depending on the cancer type. Thus, assessment of insurance expansion on outcomes for young patients with testicular cancer warrants investigation, particularly since these patients present at young ages.**

2) Results – The results are nicely presented in tables and sub-headings. Line 198, figure 1 is not visible, and the figure in line 250 is not listed here.

**Reply**

We have doubled-checked the formatting requirements for PLOS One and we can confirm that we are asked to not append the actual figures in the body of the manuscript, just the figure legends. We have uploaded the figures which should be available to the reviewer as a download at the PLOS One website.

3) Discussion - Overall, the authors provided substantial evidence regarding the context of previous literature in comparison to their results. However, I would like to see more focus in the second paragraph regarding the impact of ACA-DCE and Medicaid expansions on increased insurance coverage on cancer outcomes. Was there only one study regarding increased insurance coverage on cancer outcomes? If so, was there any additional information on why higher insurance rates did not change outcomes for the study presented?

**Reply**

We agree this paragraph is relevant in that it sets the tone for the novelty of the findings related to testicular cancer in this study. Thus, we have expanded this paragraph and the introduction to the paragraph following that one. In particular, we show that insurance expansion had differential effects on newly diagnosed breast and prostate cancer thus warranting assessment of individual tumor types. This is particularly important for testicular cancer given most men with testicular cancer are diagnosed at a young age and before age 65.

**Line 289**

The ACA-DCE and Medicaid expansions have prompted an examination of the effects of increased insurance coverage on cancer outcomes. There has been a significant decrease in the percentage of uninsured, non-elderly patients with newly diagnosed cancer following implementation of the ACA in both Medicaid expansion and non-expansion states, but with greater magnitude in Medicaid expansion states.^4, 5^ **A more recent study showed higher insurance rates did not result in changes in time to treatment for young women with breast cancer^2^. Notably, though, among patients with newly diagnosed prostate cancer, Medicaid expansion was associated with decreases in the proportion of patients presenting with high-risk disease.^3^ Thus, recent insurance expansions may differentially impact patients with different cancer.**

**Because testicular cancer is ordinarily diagnosed at a relatively young age,^6^ insurance expansion via the ACA-DCE and Medicaid expansion had substantial potential to positively impact patients with newly diagnosed testicular cancer.** According to recent reports, young men aged 20-34 have the highest incidence of being both uninsured and diagnosed with testicular cancer.^7-9^

Sincerely,

Shilajit D. Kundu, MD

676 N. St Clair St.

Arkes 23^rd^ floor

Chicago, IL 60611
312.503.5359 office
312.908.7275 fax

[Shilajit.Kundu@nm.org](mailto:Shilajit.Kundu@nm.org)

**References**

1. Sommers BD, Buchmueller T, Decker SL, Carey C, Kronick R. The Affordable Care Act has led to significant gains in health insurance and access to care for young adults. Health Affairs. 2013;32: 165-174.

2. Han X, Zhao J, Ruddy KJ, Lin CC, Sineshaw HM, Jemal A. The impact of dependent coverage expansion under the Affordable Care Act on time to breast cancer treatment among young women. PLoS ONE. 2018;13: e0198771.

3. Weiner AB, Vo AX, Desai AS, Hu JC, Spratt DE, Schaeffer EM. Changes in prostate-specific antigen at the time of prostate cancer diagnosis after Medicaid expansion in young men. Cancer. 2020;126: 3229-3236.

4. Agarwal A, Katz AJ, Chen RC. The Impact of the Affordable Care Act on Disparities in Private and Medicaid Insurance Coverage Among Patients Under 65 With Newly Diagnosed Cancer. Int J Radiat Oncol Biol Phys. 2019;105: 25-30.

5. Han X, Zang Xiong K, Kramer MR, Jemal A. The Affordable Care Act and Cancer Stage at Diagnosis Among Young Adults. J Natl Cancer Inst. 2016;108.

6. Bleyer A, Barr R, Hayes-Lattin B, et al. The distinctive biology of cancer in adolescents and young adults. Nat Rev Cancer. 2008;8: 288-298.

7. Howlader N NA, Krapcho M, Miller D, Brestt A, Yu M. SEER Cancer Statistics Review (CSR) 1975-2016. Bethesda, MD: National Cancer Institute, 2019.

8. Berchick E. Who Are the Uninsured? Most Uninsured Were Working-Age Adults [serial online] 2018. Available from URL: <https://www.census.gov/library/stories/2018/09/who-are-the-uninsured.html> [accessed July 10, 2019].

9. Cantor JC, Monheit AC, DeLia D, Lloyd K. Early impact of the Affordable Care Act on health insurance coverage of young adults. Health Serv Res. 2012;47: 1773-1790.
